# An Empirical Explanation of the Speed-Distance Effect

**DOI:** 10.1371/journal.pone.0006771

**Published:** 2009-08-26

**Authors:** William T. Wojtach, Kyongje Sung, Dale Purves

**Affiliations:** Center for Cognitive Neuroscience, Department of Neurobiology, Duke University, Durham, North Carolina, United States of America; Lund University, Sweden

## Abstract

Understanding motion perception continues to be the subject of much debate, a central challenge being to account for why the speeds and directions seen accord with neither the physical movements of objects nor their projected movements on the retina. Here we investigate the varied perceptions of speed that occur when stimuli moving across the retina traverse different projected distances (the speed-distance effect). By analyzing a database of moving objects projected onto an image plane we show that this phenomenology can be quantitatively accounted for by the frequency of occurrence of image speeds generated by perspective transformation. These results indicate that speed-distance effects are determined empirically from accumulated past experience with the relationship between image speeds and moving objects.

## Introduction

**Figure 1:**
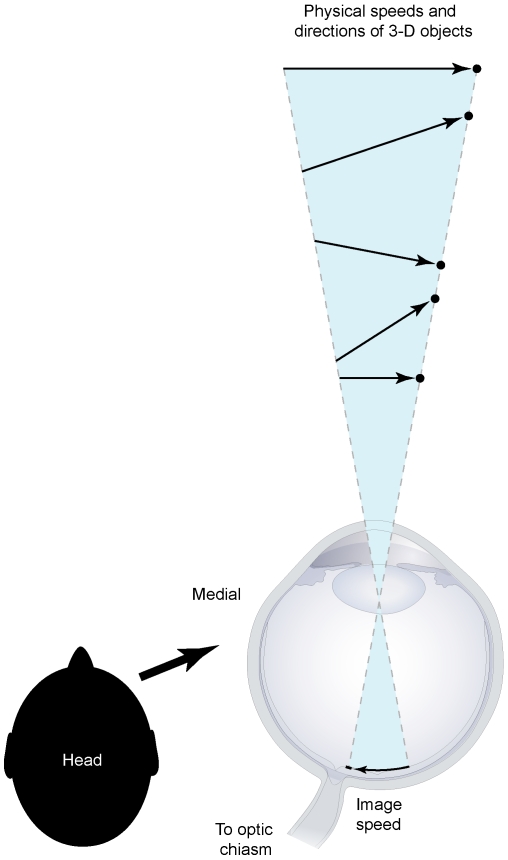
The inverse optics problem and the speed of moving objects. Due to perspective transformation, an infinite number of objects (black dots) at various distances and moving in different trajectories with different speeds (arrows) in 3-D space all generate the same 2-D image speed. Therefore, a moving image cannot specify the speeds of real-world sources (After Wojtach et al., 2008).

To succeed in their environments, humans and other visual animals must generate perceptual responses appropriate to the objects that give rise to sensory stimuli. As has long been recognized, however, the transformation of three-dimensional (3-D) space into two-dimensional (2-D) projections on the retina means that an image cannot uniquely specify its source, and therefore that visual perception cannot be determined simply by encoding the physical characteristics of retinal stimuli. This quandary is referred to as the inverse optics problem (Figure 1). Consequently, it has been appreciated for at least a century that the visual system must in some way use past experience to promote successful visual behavior [1].

Recent work on the flash-lag effect [2] and the aperture effect [3] has suggested that to contend with the inverse optics problem in the context of motion, the visual system has evolved to generate percepts empirically—i.e., according to the relative frequency of retinal images accumulated in past experience. In light of this evidence, we investigated the variable perception of speed that occurs when stimuli traverse different projected distances on the retina (the speed-distance effect). Thus, when two objects generate images with the same speed traversing different projected distances, the perception of speed elicited by such stimuli is different; conversely, when objects produce images with different speeds traversing different projected distances, the perception of speed can be the same. In keeping with earlier evidence [2], [3], we hypothesized that this effect is determined by the frequency of occurrence of image speeds traversing different projected distances in accumulated experience. Perceiving motion in this way would allow observers to produce generally successful visual responses toward objects whose actual motions cannot be derived in any direct way from retinal stimuli alone (see Figure 1).

To test this hypothesis, we used a computer-simulated environment that accurately represented the perspective transformations between moving 3-D objects and their image speeds and directions, these data serving as a proxy for the link between images and their sources that would be extracted behaviorally over time [2]. In this way, we could determine the frequency with which 3-D objects generated different image speeds traversing a range of projected distances. If the speed-distance effect indeed arises from an empirical strategy of vision, then the relative occurrence of image speeds traveling over different projected distances on the image plane should accurately predict the responses of observers in psychophysical testing.

## Materials and Methods

### Ethics statement

Informed written consent for the human psychophysical testing described below was obtained as required by the Duke University Institutional Review Board.

### The virtual environment

**Figure 2:**
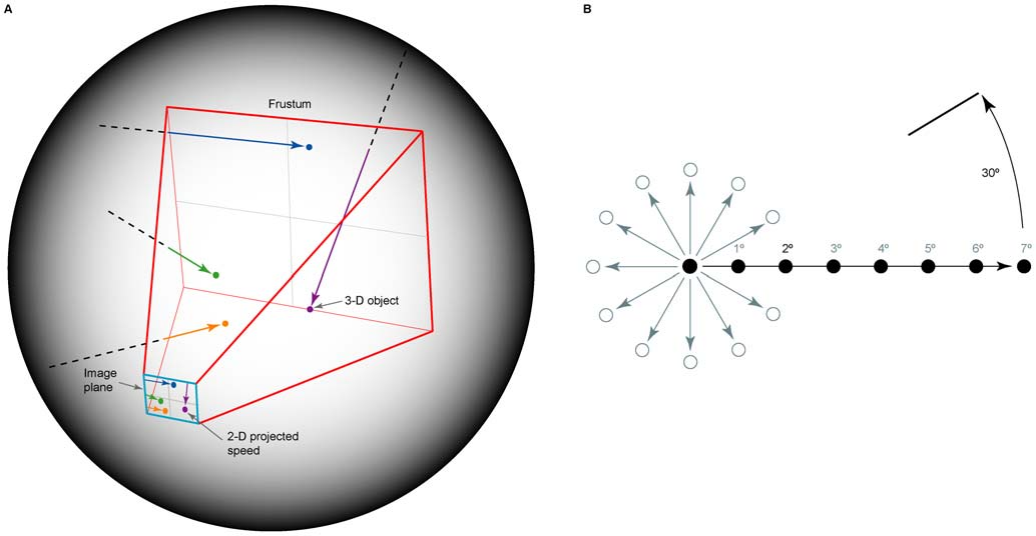
The virtual environment and sampling templates. (A) The frustum (red outline) embedded in a larger spherical space; moving 3-D objects in the frustum projected different speeds onto the image plane (blue outline), as indicated. (B) Example of the templates used to sample the image plane. For distances from 1° to 7° on the projection surface (2° in this example), image speeds from 0.1°/s to 150°/s were sampled by systematically moving the template (filled circles) to tile the entire image plane. This procedure was repeated for different orientations of the template at 30° increments, as indicated by the unfilled circles.

To obtain data about the frequency of occurrence of images needed to predict the speed-distance effect, we situated a frustum in the center of a spherical virtual environment to approximate the process of retinal image formation (Figure 2A; see [2] for details). Space within the environment was defined in uniform arbitrary units. The image plane measured 50 units in both azimuth and elevation, and was positioned at a distance from the apex of the frustum such that one square degree of visual angle corresponded to one square unit on the projection surface, resulting in a 50°×50° visual field.

Since introducing objects directly into the frustum would bias their distribution, object movement was initiated outside the frustum within the uniform space of the spherical environment. Point objects were set in motion in this space, each with a direction vector and speed assigned randomly from uniform distributions (the real-world distribution of object speeds and directions are not known; see Discussion). While the use of point objects precluded an analysis of size-distance relationships, it preserved the association between speed and distance inherent in perspective projection. Omitting size-distance effects from the statistical analysis was also in accord with the psychophysical stimuli we used, which did not include effects of distance on size (see below). When an object reached the boundary of the virtual environment a new object was randomly generated to take its place.

The distribution of object speed ranged from 0.1 to 200 units per second, giving rise to image speeds within the boundaries of human motion perception (∼0.1° to ∼150°/s) [4]. In this way, we generated more than 2.4 million objects in the environment, approximately 624,000 of which entered the frustum volume and projected onto the image plane as proxies for motion stimuli; the other 1.7 million objects did not enter the frustum, and therefore did not create projections.

### Determining the frequency of occurrence of image speeds and distances

The probability distributions of image speeds and projected distances were determined by systematically analyzing the entire image plane of the frustum at 30° increments over 360° with templates configured to correspond with the distances traversed by stimuli in the psychophysical testing (1°–7°; see Figure 2B). The projections were sampled at a resolution of 0.1 unit, or ∼6 minutes of visual arc. The slight discrepancy in angular measurement on the image plane that occurred with eccentricity from the negative z-axis was accounted for by appropriate scaling of the templates (never more than 0.18 units). Because linearly constant motion in 3-D space does not produce linearly constant 2-D projections, average image speed between the sampling points of the template was calculated. Objects whose projections satisfied distances of 1°–7° and speeds of 0.1°/s–150°/s on the image plane were then compiled to represent the accumulated relationship between moving images and their sources. These procedures yielded approximately 598,000 valid samples that were then used to compute the relevant probability distributions reported in the Results. Although a combination of factors determined a valid sample, the parameters of the sampling templates were the primary constraint on the data collected from the image plane; other features of the simulation—e.g., the speed and trajectory of objects, the length of time an object took to travel through the frustum—affected the data only indirectly.

### Psychophysical testing

Six adults (2 female; ages 18–69 years) with normal or corrected-to-normal vision participated in the psychophysical study (the authors and three participants naïve to the purposes of the experiment). Ten sessions lasting ∼30 min each were needed to complete the full range of testing.

**Figure 3:**
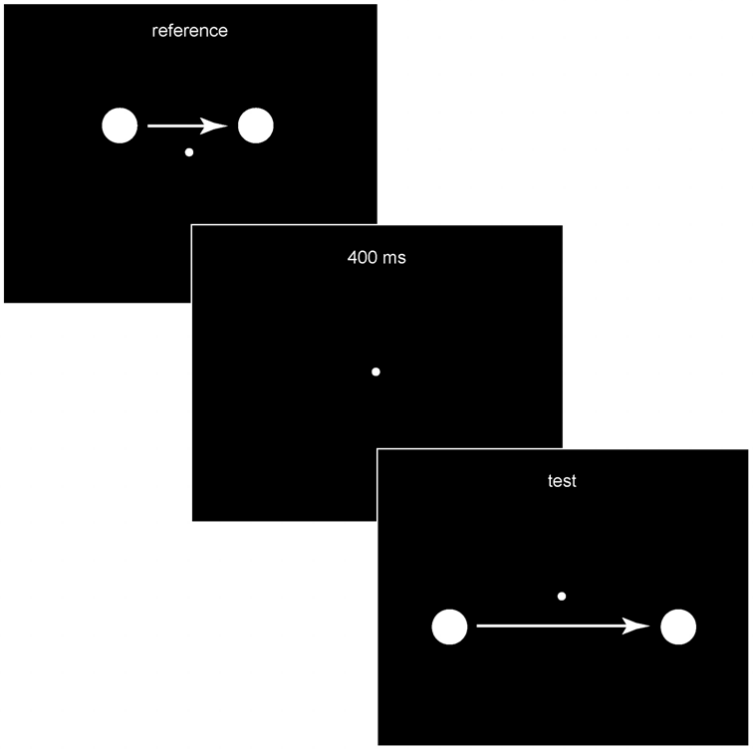
Psychophysical determination of perceived motion. Presentations of reference and test stimuli were separated by 400 ms. The distances traversed by the reference stimulus were 2°, 4°, or 6° of visual arc; the distances traversed by the test stimulus were 1°, 3°, 5°, or 7°. Observers adjusted the speed of the test stimulus, indicating when its speed appeared to be equal to the speed elicited by the reference stimulus in a random double staircase procedure (see Materials and Methods).

The perceptual functions were derived using a matching paradigm in which the speeds of two moving objects (the reference and test stimuli) were compared (see Figure 3). The stimuli were digitized using MATLAB 7.1 Psychophysics Toolbox [5] on a Dell Dimension E510 computer, and were presented as white disks (170 cd/m^2^) subtending 0.3° of visual arc on a black background. The moving objects appeared centrally on a Sony FD Trinitron 21″ CRT monitor at a frame rate of 100 Hz in a darkened testing room; a 4-pixel central fixation point was present throughout each trial. Participants viewed the stimuli binocularly with their heads stabilized by a chin rest from a distance of 140 cm. The output file for each block of trials was analyzed using JMP statistical software (v.6.0, SAS Institute), and graphed using Microsoft Excel (v.11.1.1 for Macintosh, Microsoft Corp.).

The psychophysical tests were given as a three-alternative, forced choice task, arranged in randomized double staircase format [6]. As illustrated in Figure 3, observers were first presented with a reference stimulus moving left-to-right, followed 400 ms later by a test stimulus, also moving left-to-right and centered on the fixation point. All pairs of reference and test stimulus distances were randomly presented. In any given trial, the reference stimulus traversed 2°, 4°, or 6° of visual arc at a constant speed of 2.6°/s, 3.9°/s, 6.5°/s, 7.8°/s, or 10.4°/s; the test stimulus traversed 1°, 3°, 5°, or 7° of visual arc at a constant speed that was initially either faster than or slower than the speed of the reference stimulus, depending on the staircase. Observers indicated by a keystroke whether the test stimulus generated a percept that appeared to move faster than, slower than, or the same as the speed elicited from the reference stimulus.

Depending on the response, the speed of the test stimulus either decreased (for the “faster” response) or increased (for the “slower” response) in steps of 0.65°/s in the next presentation of that particular staircase. When two consecutive “same speed” responses were reported for each staircase, the image speed of the test stimulus was recorded. These values were averaged to derive a psychophysical function for each participant. The similarity of responses across participants allowed the data to be merged. It was apparent from these results that observers were responding to the speed elicited by stimuli, and not the duration of presentation. If the responses had been based on the duration of presentation, then two stimuli with the same duration (e.g., a slower moving reference stimulus traversing 2° of arc and a faster moving test stimulus traversing 5° of arc) would have generated equivalent percepts. In agreement with findings reported by McKee [7] and Orban et al. [8], such responses were not observed.

## Results

### Deriving probability distributions from the virtual environment

To assess the hypothesis that the speed-distance effect is determined by the frequency of occurrence of image speeds over various projected distances, we first established how images traversing 1°–7° on the image plane were related to moving objects in the 3-D virtual environment. Since the environment accurately modeled perspective projection (see Figure 2A), objects traveling at different distances in depth but at the same speed generated projections that traversed different distances with different speeds on the image plane; similarly, objects traveling at different depths and speeds could project the same distance and speed on the image plane (see Figure 1).

**Figure 4:**
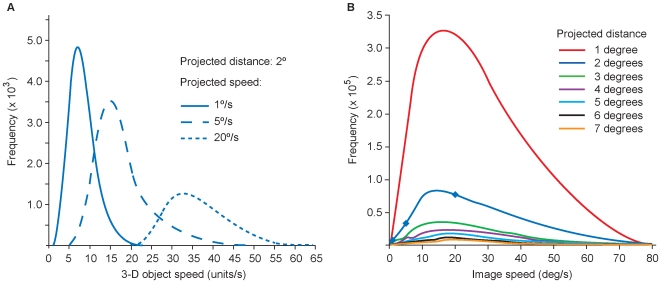
Image-source relationships derived from the virtual environment. (A) The physical speeds of 3-D objects generate a range of projected speeds over a given distance on the image plane (2° in the examples shown). As a consequence of perspective projection, relatively slow image speeds (e.g., 1°/s; solid line) are generated by objects moving at relatively slow physical speeds in 3-D space; somewhat faster image speeds (e.g., 5°/s; dashed line) arise from a wider distribution of objects in 3-D space with a larger range of physical speeds; relatively fast image speeds (e.g., 20°/s; dotted line) tend to be generated by an even wider distribution of objects moving at still greater physical speeds. Note that any given image speed can only be produced by objects moving with speeds equal to or greater than the stimulus, explaining the biased frequency distributions in (B). (B) The overall frequency distribution of image speeds generated by empirical sampling. The diamonds on the 2° projected distance function indicate the summed data from each of the three specific distributions in (A).

As an example of how we determined the relationship between ambiguous image sequences and their moving 3-D sources, consider the frequency of occurrence of image speeds traversing 2° on the image plane. By repeatedly sampling the full extent of the image plane with a 2° template (see Figure 2B), the distribution of object speeds and trajectories in 3-D space that could have produced image speeds traversing this distance was determined (Figure 4A). By compiling these data, we were able to determine how often 3-D sources generated projected speeds that traversed a distance of 2° on the image plane. Frequency distributions for projected distances from 1°–7° were acquired using this method (Figure 4B).

### Generating cumulative probability distributions

**Figure 5:**
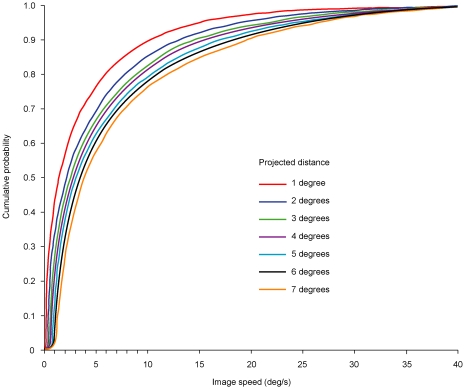
Cumulative probability distributions derived from the analyses of objects moving in the simulated environment. By transforming the frequency distribution of projected images obtained in the virtual environment (see Figure 4), the cumulative distributions order how often objects in 3-D space produced images of different speeds over different projected distances. These functions provide the basis for predicting the motion observed in psychophysical testing (see Figures 6 and 7).

The frequency distributions for projected distances of 1°–7° (Figure 4B) were then normalized and re-plotted as cumulative probability distributions (Figure 5). Ordering the data in this way indicated how often moving objects with different velocities in 3-D space underlie projected image speeds traversing different projected distances. These cumulative probability distributions thus provide a normalized empirical scale of image speeds, each point along a given distribution in Figure 5 showing the percentage of possible physical sources that generated projections equal in speed or slower than the stimulus in question.

**Figure 6 pone-0006771-g006:**
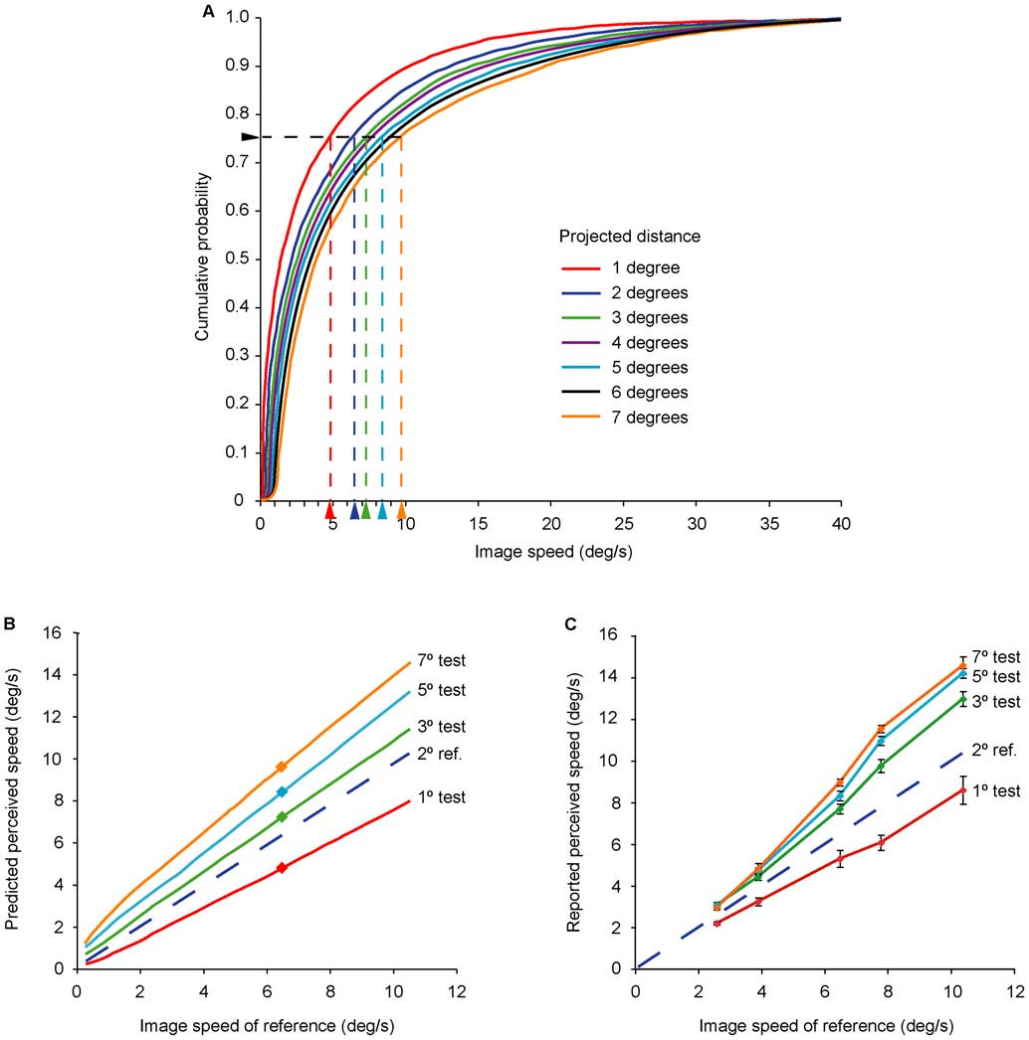
Predicting the psychophysical results elicited by image sequences traversing different distances on the image plane at different speeds but having the same percentile rank. (A) A 2° reference stimulus with an image speed of 6.5°/s (dark blue arrowhead on the abscissa) has a percentile rank at the 76^th^ percentile (black arrowhead on the ordinate). If our hypothesis of motion perception is correct, then test stimuli of 1°, 3°, 5°, and 7° with the same rank should generate perceptions of the same speed, despite their different actual speeds on the image plane (indicated by the other colored arrowheads along the abscissa). (B) The cumulative distribution data from (A) are re-plotted to indicate the predicted motion percepts elicited by the various test stimuli matched to a 2° reference stimulus as a function of the reference image speed. (C) Psychophysical functions produced by the 6 observers for test stimuli relative to the speed of the 2° reference stimulus (dashed blue line). The perceived speed reported for each test stimulus is plotted as a function of the image speed of the reference stimulus, as in (B). Bars indicate±1 standard error.

**Figure 7:**
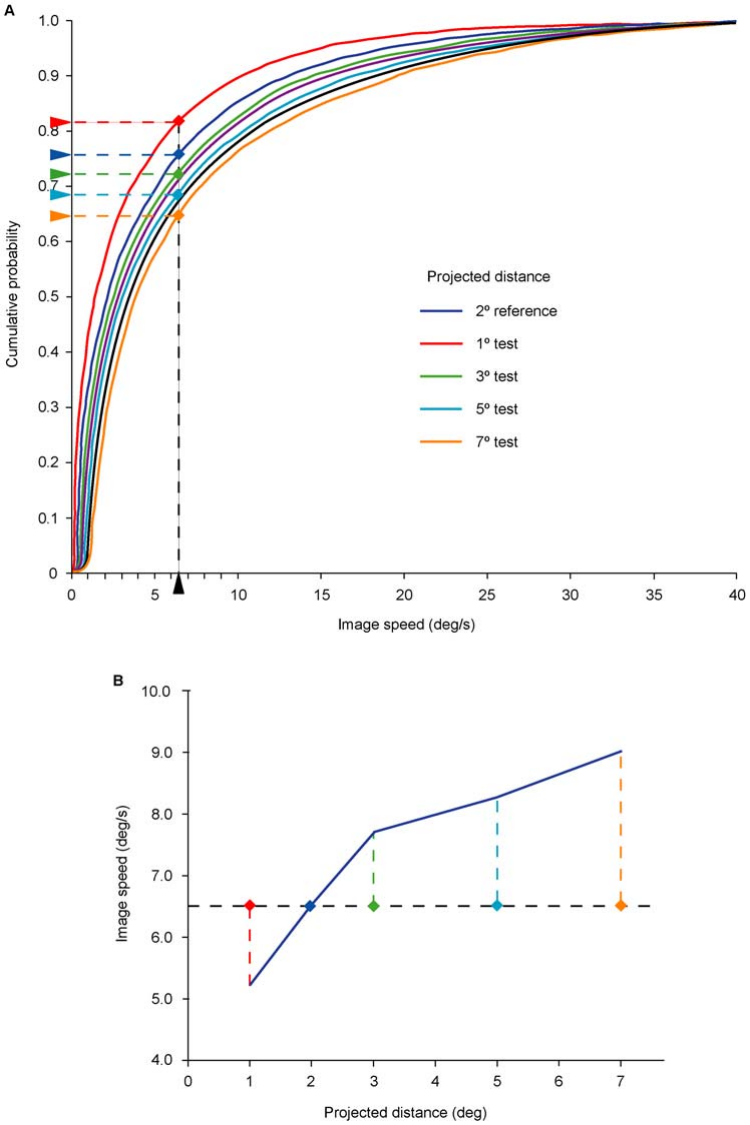
Predicting the psychophysical results elicited by image sequences traversing different distances on the image plane at the same speed but having different percentile ranks. (A) Stimuli moving across different distances on the image plane at a particular speed have different percentile ranks. For example, test stimuli traversing the image plane at a speed of 6.5°/s (black arrowhead on the abscissa) have ranks that range from the 65^th^ to the 82^nd^ percentile (colored arrowheads on the ordinate). If motion percepts are generated empirically, then the same image speed should be perceived as slower when traversing distances of 3°, 5°, or 7° in comparison with a 2° reference, but faster when traversing a test distance of 1°. (B) The blue curve indicates the projected speeds at which test stimuli traversing different projected distances (1°, 3°, 5°, and 7°) appeared the same to observers as an image speed traversing 2° at 6.5°/s (data re-plotted from Figure 6C). The area below the curve represents image speed-distance combinations perceived as slower than the reference stimulus, whereas the area above the curve represents combinations perceived as faster than the reference stimulus. Thus, test stimuli presented at 6.5°/s (dashed horizontal line) traversing distances of 3°, 5° or 7° (dashed vertical lines) are seen as moving more slowly than a 2° reference stimulus traveling at the same speed, whereas test stimuli traversing 1° at 6.5°/s are seen as moving faster.

### Predicting the speed-distance effect using percentile rank

If the speed-distance effect is determined by accumulated experience, then the corresponding percepts should be predicted by the percentile rank (cumulative probability×100%) of any given image speed when presented as a visual stimulus. In this conceptual framework, the higher an image speed ranks on the scale of accumulated image-source relationships, the faster the motion perceived. This approach therefore predicts that when two moving objects generate images that translate different projected distances at different speeds but nonetheless have the same percentile rank, their perceived speeds should be the same. Conversely, when two objects generate the same rate of image translation over different projected distances, but the cumulative probabilities of their possible sources have different percentile ranks, the perception of their speeds should be different.

We therefore assessed whether the cumulative probability distributions of image speeds in Figure 5 accurately predicted the psychophysical functions derived from observers viewing similar motion stimuli (see Figure 3). As described in Materials and Methods, observers compared the apparent speed generated by a reference stimulus with the apparent speed generated by a test stimulus traversing the different projected distances assessed in Figure 5; their task was to indicate when the speed elicited by the test stimulus appeared to match the speed elicited by the reference stimulus in a randomized double staircase procedure.

We first examined the accuracy of the percepts predicted on this basis when the reference and test stimuli had different image speeds but the same percentile rank. Consider, for example, a reference stimulus traversing 2° on the image plane at a speed of 6.5°/s compared with test stimuli traversing distances 1°, 3°, 5°, or 7° (Figure 6A). When the frequency of occurrence of image speeds of test stimuli has the same percentile rank as the reference stimulus, the frequency of occurrence of physical sources generating these image speeds is, by definition, the same. Thus, as indicated by the vertical dashed lines in Figure 6A, when an object with a projected image speed of 6.5°/s traverses 2° on the retina, test stimuli that traverse projected distances of 3°, 5°, and 7° at the same percentile rank have always had image speeds that are progressively greater than 6.5°/s; conversely, a test stimulus with a projected distance of 1° at the same rank has always had an image speed less than 6.5°/s. If the hypothesis concerning the speed-distance effect is correct, then these empirical functions should predict the relative speeds seen by the observers.

As shown in Figure 6B-C, the predictions made on this basis are in close agreement with the observed results. Thus, a reference stimulus traversing 2° at an image speed of 6.5°/s is correctly predicted to elicit the same apparent speed as stimuli with greater image speeds translating over distances of 3°, 5°, and 7°. Conversely, the same reference stimulus is correctly predicted to elicit the same apparent speed as a slower stimulus translating over 1°. Based on the sum of squared errors, the empirical functions explain>92% of the variance in the psychophysical data (1° test: 95.5%; 3° test: 95.1%; 5° test: 95.6%; 7° test: 92.5%). Similarly accurate predictions were made for reference stimuli of 4° and 6° (see Figure S1).

The cumulative probability distributions in Figure 5 also predict the phenomenology elicited by stimuli having the same image speed but traversing different projected distances (Figure 7). Consider, for instance, the different percentile ranks generated by an image speed of 6.5°/s traversing distances of 1°, 2° 3°, 5°, and 7° on the retina (Figure 7A). If relative rank determines the speed-distance effect, then stimuli moving over different projected distances with the same image speed should appear to be moving at different speeds. Thus, when a projected image is moving at 6.5°/s, the motion perceived should appear slower when traversing 2° than when traversing 1°; conversely, the same image speed should appear faster when traversing 2° than when traversing larger projected distances (e.g., 3°, 5° and 7°). As shown in Figure 7B, these predictions are also borne out.

In short, the percentile rank of image speeds in Figure 5 accurately predicts the anomalies of perceived speed that define the speed-distance effect.

## Discussion

The ability to predict the psychophysical functions in Figures 6 and 7 supports the hypothesis that the basis for the speed-distance effect is accumulated experience with image speeds and their projected distances. When this evidence is combined with the success of an empirical framework in predicting other puzzling aspects of motion perception [2], [3], as well as perceptions of brightness, color, and geometric form [9]–[11], a different concept of visual experience and its underlying mechanism emerges.

### Explaining motion perception empirically

The rationale for this concept of perception stems from the fact that a moving stimulus cannot be directly linked to the speed and trajectory of a 3-D object (see Figure 1); thus, relying on retinal images alone to generate perceptions of motion could not elicit biologically useful visual behavior. Although counterintuitive, our results demonstrate that the motion we see is better understood in terms of the frequency of occurrence of a particular retinal stimulus relative to all other moving stimuli that have occurred in past experience. In this framework, perceived motion would be determined by linking retinal stimuli with moving objects according to the relative success of behavior over evolutionary and individual time. By accurately modeling the relationships between moving objects and the perspective projection of their corresponding images, the simulated environment served as a proxy for the relative success of visual behavior instantiated in visual circuitry.

A corollary is that the motion percepts elicited from retinal stimuli correspond to neither the most likely physical speed and trajectory of an object, nor the properties of the stimulus itself, but to the locus (percentile rank) of a stimulus in accumulated past experience. In these terms, the discrepancies between the measured properties of a retinal stimulus sequence and perceived motion that define the speed-distance effect are simply a signature of an empirical strategy that evolved to contend with the inverse problem.

### The virtual environment

Although there is at present no method for obtaining empirical information about the relationships between moving images and their possible sources in the real world, the simplicity of the virtual environment we used naturally raises questions about its adequacy as a proxy for human experience with moving objects (points in this case). For example, unlike natural objects, the objects in our simulated world are all the same, and key features such as object interactions, gravity, occlusion, and many other factors could not be incorporated (see Supporting Information S1). In addition, the model is noise-free, and does not include the stochastic variability that characterizes the biological generation of retinal images [12].

A further concern is the distribution of object directions and speeds employed in the simulation. Because there is at present no empirical information available about the distribution of object vectors in the real world, we randomly assigned vectors to 3-D objects from uniform distributions of direction and speed. Since objects in space can move in any direction, a uniform distribution of directions seemed a reasonable first approximation from which to obtain the cumulative probability distributions of the variety of natural motion stimuli that would have been encountered by observers (although gravity and other factors would of course affect the distribution in nature; see above). Similarly, because the distribution of speeds in the natural world is not known, we used a uniform distribution of 3-D speeds that generated projected speeds at approximately the speeds of retinal stimuli that elicit human motion percepts (0.1°/s–150°/s; see Materials and Methods). To test these assumptions about 3-D speed, we also generated data using an asymmetric normal speed distribution (mode = ∼35 units/s), and a symmetric normal speed distribution (mode = ∼75 units/s). Although it might seem that each distribution should result in markedly different image speed distributions (and therefore different predictions of the speed-distance effect), the projections arising from these different 3-D speed distributions were generally similar, all being skewed towards slower image speeds (see Figure S2). These additional data indicate that the distribution of 3-D speeds is not the basis for the data we collected.

Given the ability to calculate image speed distributions from *a priori* assumptions about the motion of 3-D objects [13], the question arises whether the empirical data we extracted from the simulation could have been computed simply from geometrical principles alone. Because many different combinations of speeds and trajectories through space could produce the same distribution of projected speeds and distances, contending with the inverse problem (Figure 1) depends on associating 2-D images with 3-D objects over time. Since this information cannot be captured from a computed distribution of images, we adopted the more biologically relevant approach of simulating the experience of visual animals when linking 2-D projected stimuli with the perspective transformation of 3-D moving objects. This method therefore modeled more than simply the distribution of images that could be computed from 3-D objects; it also modeled the relationships between images and moving objects that the relative success of behavior would extract over time.

### Explaining the biases in the cumulative probability distributions

Despite the limitations of the simulation, the predicted functions we derived are in good agreement with the psychophysical results. The reason is that the principles of perspective projection, which the simulation captures nearly perfectly, are the major determinant of the image-source statistics pertinent to perceived motion. The only potential concern in this regard is the design of the frustum, which necessarily affects the ability of objects to project images. The dimensions of frustum were therefore created to enable a range of projected speeds that mimic those normally experienced by humans (∼0.1°/s–150°/s).

Perspective projection also explains why the cumulative probability distributions of image-source relationships have the shape they do, and thus how they influence the percentile rank of motion stimuli (see Figures 5–7). The projected distances of moving stimuli in either the simulated or real world will always be equal to or shorter than the 3-D distances traveled by objects; in consequence a greater number of image sequences are experienced over smaller projected distances (see Figure 5). Furthermore, because perspective projection requires that the actual speeds of objects are always greater than or equal to their projected speeds, image speed distributions will always be weighted toward speeds slower than the range of physical speeds in 3-D space [12]–[15]; see also Figure S2). Together, these features of the projections of moving objects give rise to the non-linear biases apparent in the cumulative probabilities in Figure 5, which in turn determine the percentile rank of stimuli in past experience.

### Other models of motion processing

The relationship between the physical movement of objects, their projected images, and perceived motion has long been a puzzle, giving rise to a variety of theories about its neural basis. The prevailing physiological models of motion perception have generally been based on a processing hierarchy in which the lower-order receptive field properties of motion sensitive neurons in V1 are used to progressively construct the more complex responses of higher-order cortical regions such as those observed in areas MT and MST in the non-human primate brain and MT+ in humans, the culmination of this process being the motion perceived [16]–[22]. Although this approach has been amended with two-stage [23] or three-stage [24] processing schemes, as well as by the addition of “component cells,” “pattern cells,” and cascade models that could explain further details of motion perception [25], [26], this idea remains a popular conception of how motion percepts are generated.

In addition to physiological models of visual motion, a number of other models have been proposed. Of these, algorithmic strategies for feature-detection [13], [14], [27]–[29], spatiotemporal energy models [30]–[34], and Bayesian approaches have received the most attention. Although each of these models can explain some important aspects of motion perception, none explains the extraordinary range of anomalous motion percepts experienced by observers, including the psychophysical results we report here.

Consider, for instance, whether application of Bayesian decision theory to the simulation data would have predicted the observed psychophysical functions in the Results. The problem we addressed can be easily formulated in Bayesian terms. Since the goal of a Bayesian model is to estimate the most probable 3-D source based on the available 2-D evidence, the variables pertinent to the current study are the 3-D object speed, 2-D image speed, and 2-D image distance (recall that since the reference and test stimuli always moved horizontally from left-to-right, perceived direction does not need to be considered). These parameters can be expressed in Bayesian terms as, 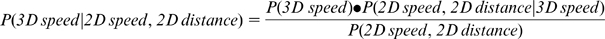 where P is probability. In this formulation, the first term on the right side of the equation, P(3D speed), is the prior probability distribution, which describes the experience of human observers with 3-D speeds. Because this distribution in the simulation was uniform, the shape of the prior is also uniform. The second term on the right side of the equation, P(2D speed, 2D distance | 3D speed), is the likelihood function, which describes the probability that a given 3-D speed will have generated any specific 2-D image speed and distance. The product of the prior and relevant likelihood function divided by a normalization constant, P(2D speed, 2D distance), generates the posterior probability distribution, P(3D speed | 2D speed, 2D distance). The posterior distribution is therefore a subset of the prior, indicating the relative probabilities of possible 3-D object speeds that could have produced a specific 2-D image sequence in question. Since in a Bayesian formulation motion percepts correspond to a particular value in the posterior distribution, a basis for choosing this value is needed. Typically, this criterion is the presumed biological usefulness of the value; under the assumption that this would be the most frequently occurring source in past experience, an index such as the mean, median, or mode of the posterior distribution is used to generate the value that determines the percept.

**Figure 8:**
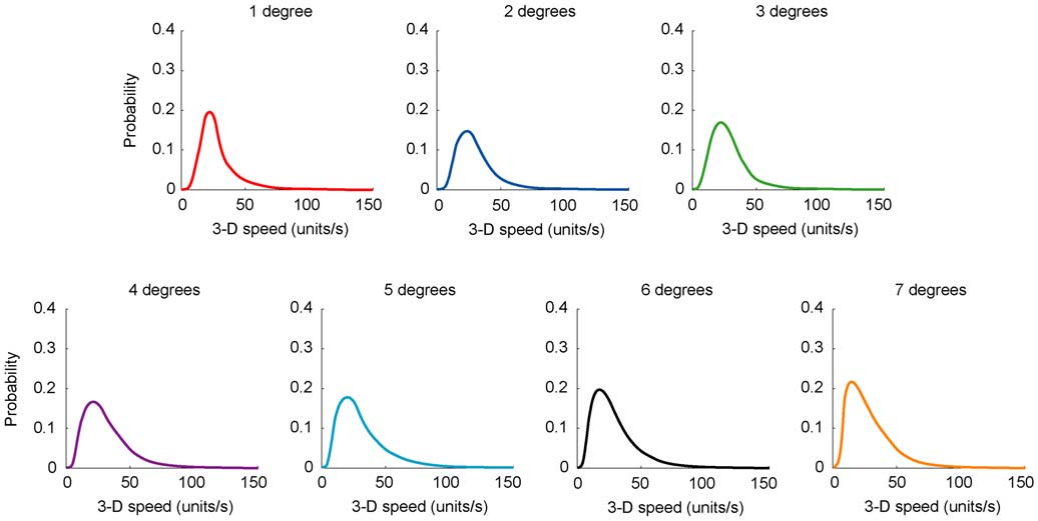
Bayesian posterior probability distributions for stimuli traversing projected distances of 1°–7° at 6.5°/s. For each projected distance from 1°–7°, the probability of 3-D speeds that can give rise to an image speed of 6.5°/s are shown. Calculating the mean, median, or mode of each distribution results in the predicted percept for the specific projected distance. Similar distributions for the other image speeds tested (2.6°/s, 3.9°/s, 7.8°/s, and 10.4°/s) were generated, but are not shown.

**Figure 9:**
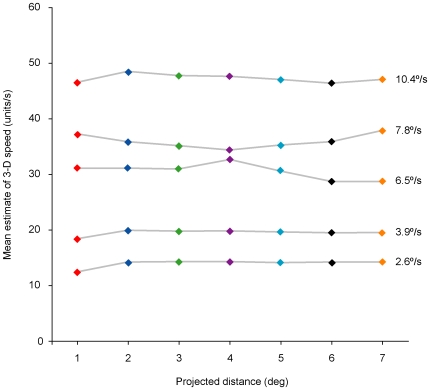
Bayesian predictions of the psychophysical results elicited by image sequences traversing different projected distances at the same speed. To predict the relevant percepts, the mean of each posterior in Figure 8 was calculated (indicated by the corresponding colored points). In contrast to the observed psychophysical results, a Bayesian model predicts little or no change of perceived motion in response to a given image speed (2.6°/s, 3.9°/s, 6.5°/s, 7.8°/s, or 10.4°/s) traversing different projected distances (1°–7°) on the image plane (cf. Figure 7B; see also Figure 6C).

To predict the psychophysical observations reported in the Results in Bayesian terms, we calculated posterior probability distributions for stimuli traversing 1°–7° of projected distance at 6.5°/s (Figure 8; posterior distributions for each of the image speeds tested in the psychophysical studies were similarly derived). As indicated in Figure 7B, the psychophysical data showed that, for stimuli traversing projected distances of 1°–7° at 6.5°/s, the perceived speed decreased progressively as the projected distance increased. In a Bayesian formulation, however, the mean of each posterior probability distribution in Figure 8 predicts that observers should perceive the speed elicited by such stimuli as being approximately the same regardless of the projected distance (Figure 9).

The reason for the different outcome generated by a Bayesian framework compared to the predictions made by empirical ranking is based in how each approach conceptualizes the goal of vision [35]. A Bayesian framework assumes that a motion percept is determined by the most likely 3-D speed that generated the stimulus, the implied goal being to link percepts with the specific physical characteristics of sources in the world underlying a stimulus sequence. As indicated in Figure 1, however, the inverse optics problem precludes direct access to the properties of the physical world, making the expressed goal of a Bayesian framework impossible to achieve as formulated. In contrast, the method of empirical ranking predicts motion percepts based on the full range of past experience rather than a particular state of the world, and these conform closely to the observed psychophysical functions.

### Further implications arising from an empirical strategy of motion perception

If the visual system has indeed evolved to link projected images with objects according to accumulated behavioral feedback, then any attempt to understand vision in terms of the properties of images alone should fail. As illustrated in Figure 1, the inverse problem implies that to be successful any account of visual perception must be based on the empirical relationships between images and their possible sources. The evidence here suggests that this information accumulates in visual system circuitry over evolutionary and individual time, giving rise to perceptions that represent the movements of objects in terms of their biological utility rather than the speeds and trajectories the objects actually have. Considered in this way, the discrepancies between the measured properties of a retinal stimulus sequence and perceived motion are due to the visual processing strategy that evolved to contend with the inverse problem.

Some recent neurobiological evidence consistent with this interpretation of vision comes from optical imaging of striate and extrastriate visual cortex. Thus, it has been shown that stimuli moving in different directions and at different speeds can elicit the same pattern of neuronal activity [34]. At the same time, there continues to be much debate over the hierarchical concept of visual cortical organization generally and the proper interpretation of striate and extrastriate processing in particular [36]–[38], These and other observations [39] are all consistent with an empirical strategy of sensory processing.

## Supporting Information

Supporting Information S1 (0.03 MB DOC)

Figure S1 Figure S1. Comparison of the functions predicted by empirical ranking with the results of psychophysical testing for reference stimuli of 4° and 6°. The presentation is similar to the illustration of the 2° results in Figure 6. (A) The cumulative distribution data from Figure 5 re-plotted to indicate the predicted motion percepts for a 4° reference stimulus as a function of image speed. (B) The psychophysical functions from the 6 subjects for a 4° reference stimulus. (C) The cumulative distribution data for a 6° reference stimulus plotted as a function of image speed. (D) The psychophysical functions from the subjects for a 6° reference stimulus. As with the 2° reference stimulus, the amount of variance explained by the simulation for 4° and 6° reference stimuli was quite good (4° reference = 1° test: 80.9%, 3° test: 99.4%, 5° test: 98.7%, 7° test: 95.9%; 6° reference = 1° test: 31.2%, 3° test: 95.3%, 5° test: 99.7%, 7° test: 98.9%). The single outlier (6° reference, 1° test) arises from small variations in the cumulative distribution at these distances, resulting in a slight downward shift of the 1° function. Smoothing the cumulative distribution corrects this anomaly; however, the uncorrected results are presented. Bars in (B) and (D) indicate±1 s.e.m.
(0.58 MB TIF)

Figure S2 Figure S2. Distribution of average image speeds generated from different 3-D speed distributions in the virtual environment. (A) Uniform speed distribution. (B) Asymmetric normal speed distribution (mode = ∼35 units/s). (C) Symmetric normal speed distribution (mode = ∼75 units/s). The prevalence of slow image speeds is primarily the result of perspective projection, and not the 3-D distribution of object speeds. See [S5] for additional information.
(0.60 MB TIF)
