# Supplementary material for: An Empirical Explanation of the Speed-Distance Effect
Source: PLoS One. 2009 Aug 26;4(8):e6771. doi: 10.1371/journal.pone.0006771 (PMC2727946; doi:10.1371/journal.pone.0006771)
Supplement: Supporting Information S1 — (0.03 MB DOC) [file pone.0006771.s001.doc]

**Supporting Information S1**

*Further comments on the limitations of the simulated environment*

In addition to those mentioned in the Discussion, the design of the simulation used has several other limitations with respect to both biology and physics that deserve comment. With respect to biological vision, the simulation data takes no account of differences in sensitivity as function of retinal eccentricity [S1-S3]. Nor does it take account of the fact that humans and other visual animals do not pay equal attention to all classes of stimuli in natural viewing, but respond to those that have particular significance for the species in question [S4]. There are also inadequacies with respect to real-world physics not mentioned in the text. For instance, we ignored friction when simulating object motion.

Given this list of limitations, one must wonder about the adequacy of the simulated data in predicting the perceptual responses to even simple motion stimuli. As noted in Materials and Methods and the Discussion, however, perspective projection is accurately represented, and projective geometry is the primary determinant of the frequency of occurrence of image speeds [S5].

**Supporting References**

S1. Johnson CA, Leibowitz HW (1976) Velocity-time reciprocity in the perception of motion: Foveal and peripheral determinations. Vision Res 16: 177-180.

S2. McKee SP, Nakayama K (1984) The detection of motion in the peripheral visual field. Vision Res 24: 25-32.

S3. Tynan PD, Sekuler R (1982) Motion processing in peripheral vision: Reaction time and perceived velocity. Vision Res 22: 61-68.

S4. Yarbus AL (1967) Eye movements and vision (Haigh B, Riggs LA, eds). New York: Plenum Press.

S5. Wojtach WT, Sung K, Truong S, Purves D (2008) An empirical explanation of the flash-lag effect. Proc Natl Acad Sci U S A 105: 16338-16343.

**Figure S1. Comparison of the functions predicted by empirical ranking with the results of psychophysical testing for reference stimuli of 4º and 6º.** The presentation is similar to the illustration of the 2º results in Figure 6. (A) The cumulative distribution data from Figure 5 re-plotted to indicate the predicted motion percepts for a 4º reference stimulus as a function of image speed. (B) The psychophysical functions from the 6 subjects for a 4º reference stimulus. (C) The cumulative distribution data for a 6º reference stimulus plotted as a function of image speed. (D) The psychophysical functions from the subjects for a 6º reference stimulus. As with the 2º reference stimulus, the amount of variance explained by the simulation for 4º and 6º reference stimuli was quite good (4º reference = 1º test: 80.9%, 3º test: 99.4%, 5º test: 98.7%, 7º test: 95.9%; 6º reference = 1º test: 31.2%, 3º test: 95.3%, 5º test: 99.7%, 7º test: 98.9%). The single outlier (6º reference, 1º test) arises from small variations in the cumulative distribution at these distances, resulting in a slight downward shift of the 1º function. Smoothing the cumulative distribution corrects this anomaly; however, the uncorrected results are presented. Bars in (B) and (D) indicate ± 1 s.e.m.

**Figure S2. Distribution of average image speeds generated from different 3-D speed distributions in the virtual environment.** (A) Uniform speed distribution. (B) Asymmetric normal speed distribution (mode = ~35 units/s). (C) Symmetric normal speed distribution (mode = ~75 units/s). The prevalence of slow image speeds is primarily the result of perspective projection, and not the 3-D distribution of object speeds. See [S5] for additional information.
