# Supplementary figures and images for: An Empirical Explanation of the Speed-Distance Effect
Source: PLoS One. 2009 Aug 26;4(8):e6771. doi: 10.1371/journal.pone.0006771 (PMC2727946; doi:10.1371/journal.pone.0006771)

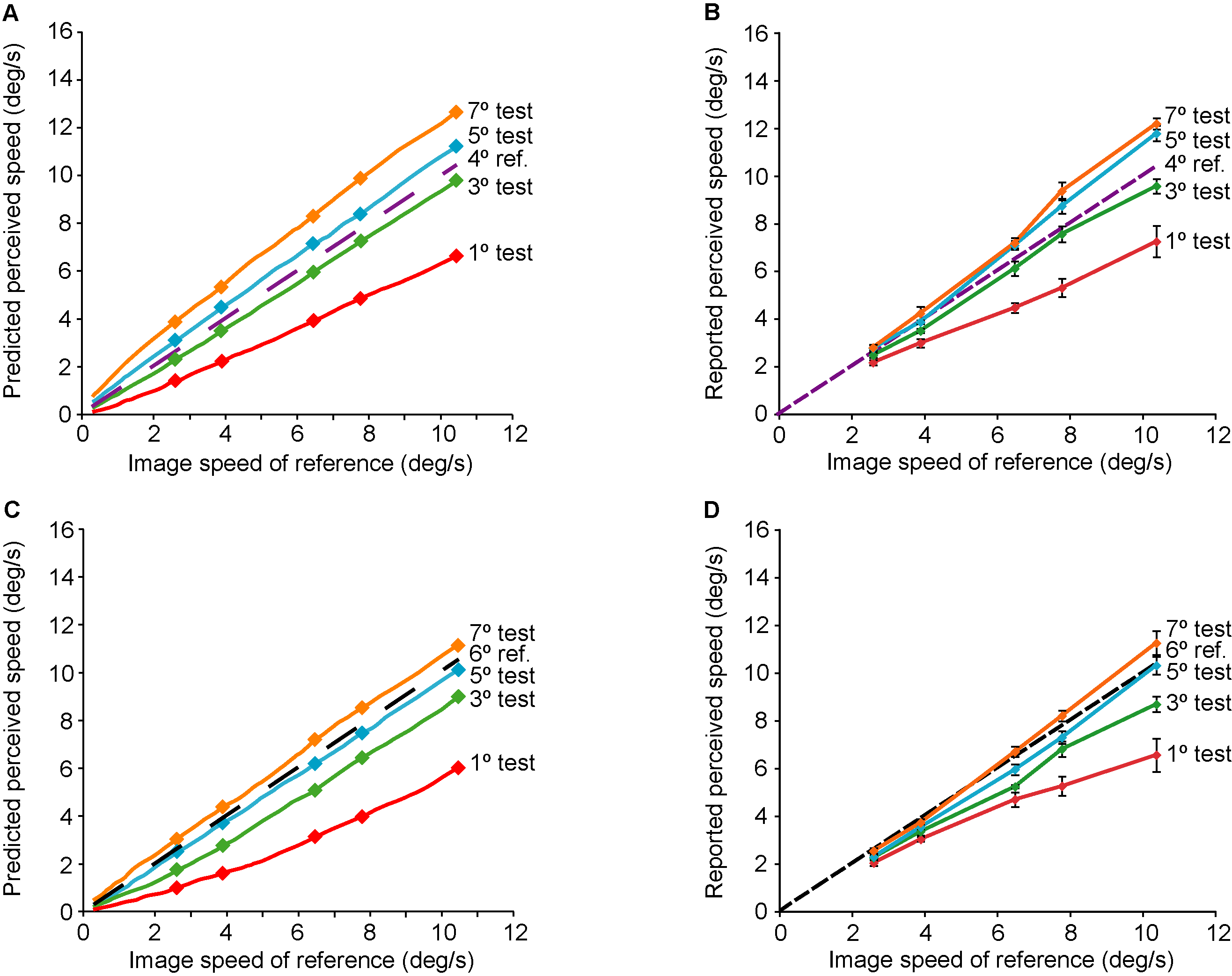

Supplement: Figure S1 — Comparison of the functions predicted by empirical ranking with the results of psychophysical testing for reference stimuli of 4° and 6°. The presentation is similar to the illustration of the 2° results in Figure 6. (A) The cumulative distribution data from Figure 5 re-plotted to indicate the predicted motion percepts for a 4° reference stimulus as a function of image speed. (B) The psychophysical functions from the 6 subjects for a 4° reference stimulus. (C) The cumulative distribution data for a 6° reference stimulus plotted as a function of image speed. (D) The psychophysical functions from the subjects for a 6° reference stimulus. As with the 2° reference stimulus, the amount of variance explained by the simulation for 4° and 6° reference stimuli was quite good (4° reference = 1° test: 80.9%, 3° test: 99.4%, 5° test: 98.7%, 7° test: 95.9%; 6° reference = 1° test: 31.2%, 3° test: 95.3%, 5° test: 99.7%, 7° test: 98.9%). The single outlier (6° reference, 1° test) arises from small variations in the cumulative distribution at these distances, resulting in a slight downward shift of the 1° function. Smoothing the cumulative distribution corrects this anomaly; however, the uncorrected results are presented. Bars in (B) and (D) indicate±1 s.e.m. (0.58 MB TIF) [file pone.0006771.s002.tif]

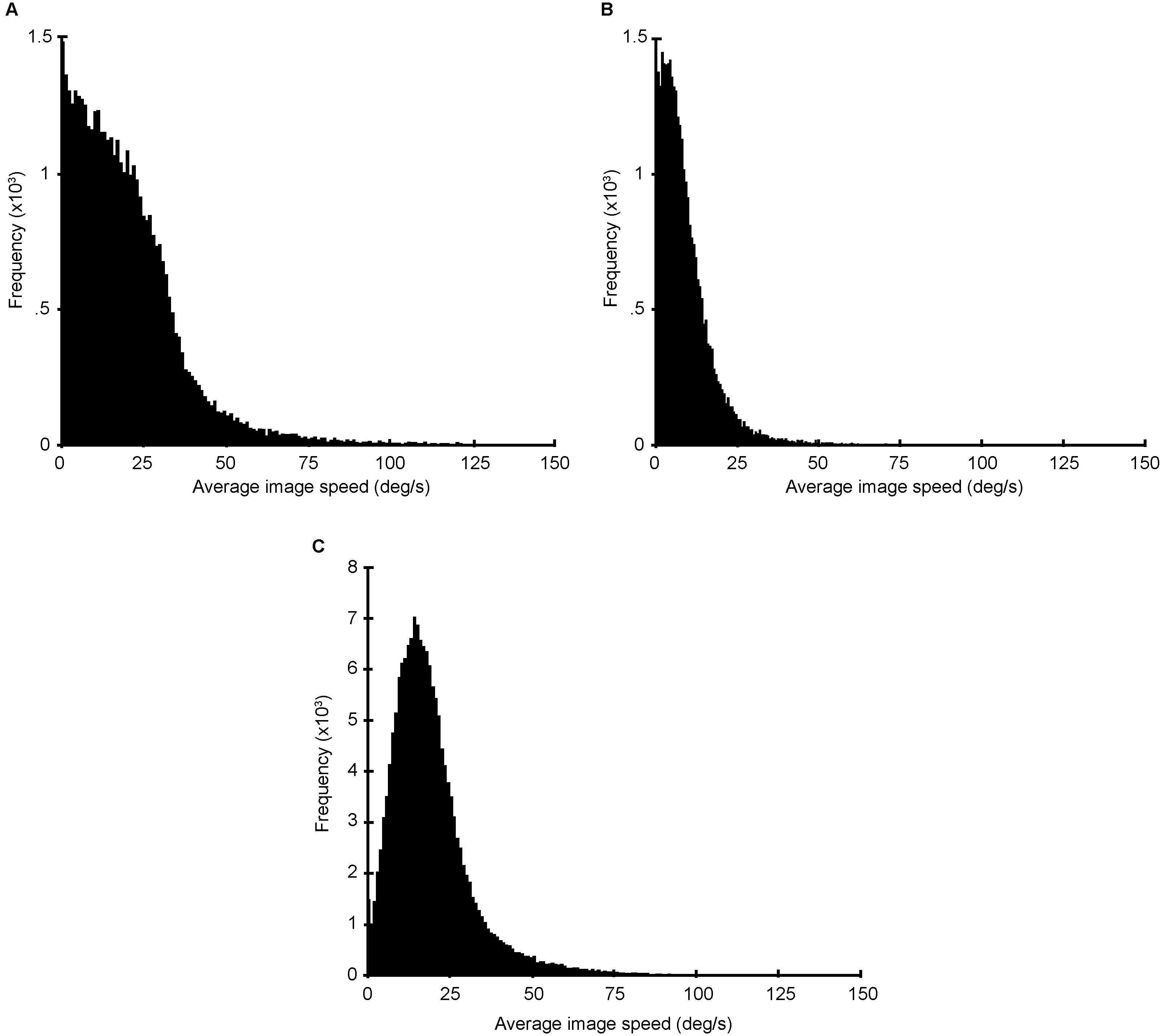

Supplement: Figure S2 — Distribution of average image speeds generated from different 3-D speed distributions in the virtual environment. (A) Uniform speed distribution. (B) Asymmetric normal speed distribution (mode = ∼35 units/s). (C) Symmetric normal speed distribution (mode = ∼75 units/s). The prevalence of slow image speeds is primarily the result of perspective projection, and not the 3-D distribution of object speeds. See [S5] for additional information. (0.60 MB TIF) [file pone.0006771.s003.tif]
